# Missed injuries in trauma patients: A literature review

**DOI:** 10.1186/1754-9493-2-20

**Published:** 2008-08-23

**Authors:** Roman Pfeifer, Hans-Christoph Pape

**Affiliations:** 1 Department of Orthopaedic Surgery, University of Pittsburgh Medical Center, Kaufmann Medical Building, Suite 1010, 3471 Fifth Avenue, Pittsburgh, PA 15213, USA

## Abstract

**Background:**

Overlooked injuries and delayed diagnoses are still common problems in the treatment of polytrauma patients. Therefore, ongoing documentation describing the incidence rates of missed injuries, clinically significant missed injuries, contributing factors and outcome is necessary to improve the quality of trauma care. This review summarizes the available literature on missed injuries, focusing on overlooked muscoloskeletal injuries.

**Methods:**

Manuscripts dealing with missed injuries after trauma were reviewed. The following search modules were selected in PubMed: Missed injuries, Delayed diagnoses, Trauma, Musculoskeletal injuires. Three time periods were differentiated: (n = 2, 1980–1990), (n = 6, 1990–2000), and (n = 9, 2000-Present).

**Results:**

We found a wide spread distribution of missed injuries and delayed diagnoses incidence rates (1.3% to 39%). Approximately 15 to 22.3% of patients with missed injuries had clinically significant missed injuries. Furthermore, we observed a decrease of missed pelvic and hip injuries within the last decade.

**Conclusion:**

The lack of standardized studies using comparable definitions for missed injuries and clinically significant missed injuries call for further investigations, which are necessary to produce more reliable data. Furthermore, improvements in diagnostic techniques (e.g. the use of multi-slice CT) may lead to a decreased incidence of missed pelvic injuries. Finally, the standardized tertiary trauma survey is vitally important in the detection of clinically significant missed injuries and should be included in trauma care.

## Background

Patients who have been severely injured in road accidents [1,2], especially those with head injury [1,3,4], a Glasgow Coma Scale (GCS) score of eight or lower [5,6], and a greater Injury Severity Score (ISS) [1-3,5-9], are more likely to have missed injuries or delayed diagnoses. The majority of treatment errors occur in the emergency department [10-12], the intensive care unit (ICU) [10,12] and the operating room [12]. Gruen et al. [10] analysed patterns of error contributing to trauma mortality in 64 trauma patients with recognized errors in care. Errors were found to occur in haemorrhage control (28%), airway management (16%), management of unstable patients (14%) and prophylaxis (11%). The authors suggest that strategies for error-reduction should be addressed in both the emergency department and intensive care unit. However, ongoing documentation describing the incidence rates of missed injuries, clinically significant missed injuries, contributing factors and outcome is necessary to improve the quality of trauma care.

This retrospective series review summarizes the available literature on missed injuries and analyzes whether changes in incidence rates of missed musculoskeletal injuries have occurred over the last three decades. We hypothesize that a decrease of incidence rates of missed injuries occurred due to improvements in treatment and diagnostics. In addition, it evaluates the circumstances that cause missed injuries and describes strategies to limit these pitfalls.

## Methods

### Literature Search

To identify the relevant publications, a Medline database search through PubMed (time period 1980 – July 2008) was performed. Relevant studies were retrieved using the following sequences of key words: Missed injuries, Delayed diagnoses, Trauma, Musculoskeletal injuries. Synonyms were used to find further relevant literature. In addition, we reviewed the references from the resulting publications to identify further potential articles to be included in our study. After Medline searches were completed, all acticles in English- and German-language and articles published after 1980 were screened for inclusion and exclusion criteria.

### Selection of Relevant Papers

#### Missed injuries

• Injuries that were not identified by primary and secondary survey. All diagnoses made in tertiary survey (24 h). [6 studies]

• Injuries ditacted after the admission to the ICU (24 h). [4 studies]

• Injuries found after complete assessment and diagnostics, and are directly related to the injury. [4 studies]

• Injuries that were missed within 6 to12 hours. [2 studies (12 hour time point) 1 study (6 hour time point)]

### Clinically significant missed injuries

• Missed injuries that are associated with high morbidity and mortality. [2 studies]

• Missed injuries that require additional procedures and alterations of therapy. [1 study]

• Missed injuries with significant pain, complications, residul disability and death.

[1 study]

#### Analysis of relevant Papers

A total of seventeen articles satisfied the inclusion and exclusion criteria for this analysis. We reviewed and summarized the findings published in the studies. Variables of interest included authors, year of publication, type of study, sample size, average age of patients in years, Injury Severity Score (ISS), percentage of patients involved in motor vihicle accidents (MVA), percentage of patients with blunt trauma, and incidence rates of missed injuries. Furthermore, missed injuries from the publications above were classified in 3 groups (minor injuries, major injuries, life threatening injuries) to assess the clinical relevance of these overlooked injuries.

#### Minor injuries

Hand, wrist, foot, ankle, forearm, uncomplex soft tissue injuries and fractures, rupture of ligaments and muscle tendons were defined as minor injuries.

#### Major injuries

Skull injuries, neurological and arterial lesions, liver, spleen, and intestinal lacerations, femoral, humeral, pelvic, and spine fractures and dislocations were defined as major inuries.

#### Life threatening injuries

Injuries of main vessels in thorax, Hemothorax and Pneumothorax were defined as life threatening injuries.

All data were summarized in tables and median velues and percantages were calculated using Excel (Microsoft Office).

## Results

We found seventeen prospective (6) and retrospective (11) publications that fit the criteria within the three decade time period. The mean study population was 1124 (Median: 709, range 65–3996). Two manuscripts analyzed data between 1980 and 1990, six between 1990 and 2000, and nine between 2000 and July 2008. For the seventeen publications, the median age was 34 points (range, 8.4–39.6), the Injury Severity Score was 17.2 points (range, 14–26), the median percentage of patients involved in motor vehicle accident was 68% (range, 46–84.6%), 92% (median) (range, 88–100%) sustained a blunt trauma, and the median percentage for musculoskeletal injuries was 69.2% (range, 4–100%).

Several studies dealing with missed injuries and delayed diagnoses have been published and report an incidence of 1.3% to 39% [1-3,5-9,13-20] (see Table 1). The mean percentage of unrecognized injuries in all studies mentioned above is approximately nine. A comparatively small number of studies have distinguished between clinically significant missed injuries and missed injuries in general [1,2,5,7](Table 2). According to these publications, 15–22.3% of patients with missed injuries had clinically significant missed injuries.

**Table 1:** Total missed injuries and contributing factors found in studies

Study	N	Population	Total missed injuries	Cause X-Ray errors	Clinical errors
Vles et al., 2003 # [3]	3.879	Trauma Patients	1,3%	X	X
Robertson et al., 1996 * [8]	3.996	Rural Area Trauma Patients	1,4%	X	X
Juhl et al., 1990 # [13]	783	Orthopaedic Department Pat.	2,2%	X	X
Born et al., 1989 # [14]	1.006	Multisystem Trauma Patients	3%	X	X
Wei et al., 2006 * [15]	3.081	Emergency Radiology Pat.	3,7%	X	-
Laasonen et al., 1991 * [16]	340	Multiple Injured Patiens	4,2%	X	-
Kalemoglu et al., 2006 * [6]	709	Major Trauma Patients	4,8%	X	X
Pehle et al., 2006 * [17]	1.187	Multiple Trauma Patients	4,9%	X	X
Kremli, 1996 *[18]	638	Trauma Patients	6%	X	X
Buduhan et al., 2000 * [5]	567	Multiple Trauma Patients	8,1%	X	X
Houshian et al., 2002 # [1]	786	Major Trauma Patients	8,1%	X	X
Chan et al., 1980 * [19]	327	Multiple Injured Patients	12%	X	X
Rizoli et al., 1994 * [7]	432	Blunt Trauma Patients	13,6%	X	X
Soundappan et al., 2004 #[20]	76	Children with missed Injuries	16%	X	X
Brooks et al., 2004 * [9]	65	Major Trauma Patients	22,2%	X	X
Janjua et al., 1998 # [2]	206	Trauma Patients	39%	X	X

N = Patients in study; Prospective study #; Retrospective study *

**Table 2:** Percentage of clinically significant missed injuries analysing all patients with missed injuries

Study	N	Pat. with clinically sign. missed injuries
Buduhan et al., 2000 [5]	567	15.2%
Houshian et al., 2002 [1]	786	15.4%
Rizoli et al., 1994 [7]	432	20.3%
Janjua et al., 1998 [2]	206	22.3%

N = Patients in study

Analysis of articles published from 1980 to 2006 (Table 3) indicated a lower incidence of missed pelvic and hip injuries from 2000 to 2006 [1-3,5-8,13,14,18-21]. According to available studies from the 1980s, all missed pelvic injury rates exceeded 10%. Out of five publications from the 1990s, one reported missed pelvic injury rates above 10% and four reported results below 10%. All publications found from 2000 to 2006 reported missed pelvic injury rates below 10%. A similar trend was not observed for lower and upper extremity injuries.

**Table 3:** Missed extremity and musculoskeletal injuries after polytrauma

Study	N	Foot/Ankle	Leg	Hip/Pelvis	Wrist/Hand	Arm	Spine
Chan et al., 1980 [19]	327	22.4%	16.3%	18.4%	10.2%	12.2%	6.1%
Born et al., 1989 [14]	1006	23.1%	10.3%	10.3%	5.1%	38.5%	12.8%
							
Juhl et al., 1990 [13]	783	23.4%	6.3%	8.5%	32.9%	10.6%	7.4%
Rizoli et al., 1994 [7]	432	8.1%	5.1%	2.7%	17.6%	16.2%	9.5%
Kremli, 1996 [18]	638	17.5%	32.5%	16.3%	6.2%	16.3%	7.5%
Robertson et al., 1996 [8]	3996	8.6%	10%	3%	11.4%	7.1%	10%
Janjua et al., 1998 [2]	206	Lower Limb:15.2%		6.5%	Upper Limb:26.6%		4.2%
							
Buduhan et al., 2000 [5]	567	Extremiries: 33.3%		7.9%	Extremiries: 33.3%		7.9%
Guly, 2001 [21]	934	25.8%	4.3%	4.9%	17.2%/21.7%	15.1%	3.4%
Houshian et al., 2002 [1]	876	12.8%	8.1%	8.1%	8.1%	11.6%	5.8%
Vles et al., 2003 [3]	3879	12.2%	6.1%	6.1%	4.1%	12.2%	8%
Soundappan et al., 2004 [20]	76	Lower Limb: 31%		-	Upper Limb: 23%		15%
Kalemoglu et al., 2006 [6]	709	Extremiries: 38.2%		9.3%	Extremiries: 38.2%		9.3%

N = Patients in study

Unrecognized injuries listed in studies were classified in three different types: (minor, major, life threatening injuries) to assess the clinical relevance (Table 4) [1,3,7,8,13,14,18,19]. Approximately 27–66% of all delayed diagnoses were major injuries. In addition, it can be seen that the most studies identified life threatening injuries. In three publications only a low percentage (1–4%) of life threatening injuries was missed.

**Table 4:** Missed injuries extracted from reviewed studies and classified in minor, major, and life threatening injuries

Study		Injuries classified in	
	minor	major	life threatening

Chan et al., 1980 [19]	51.1%	48.9%	0%
Born et al., 1989 [14]	66.7%	33.3%	0%
Juhl et al., 1990 [13]	72.3%	27.7%	0%
Rizoli et al., 1994 [7]	56.8%	41.9%	1.3%
Kremli, 1996 [18]	33.7%	66.3%	0%
Robertson et al., 1996 [8]	35.3%	60.3%	4.4%
Houshian et al., 2002 [1]	36.1%	61.6%	2.3%
Vles et al., 2003 [3]	47.3%	52.7%	0%

## Discussion

Our review demonstrates the following main findings: First, we found a wide spread distribution (1.3%–39%) of incidence rates for missed injuries and delayed diagnoses. Second, approximately 15 to 22.3% of patients with missed injuries have clinically significant missed injuries. Third, incidence rates of missed pelvis and hip injuries have decreased over the last three decades (1980-Present). Fourth, approximately 27–66% of unrecognized diagnoses in studies were major injuries.

The difference between the results of the studies indicates that the true incidence of missed injuries and delayed diagnoses is difficult to determine. A discrepancy in the definition of what constitutes a missed injury may be the major cause. Another possibility is that many authors limited their investigations to a special field of interest. Some investigators report missed injuries in multiple trauma patients [5,9,17,19], other authors describe unrecognized injuries in patients with abdominal [22] and orthopaedic trauma [13,14,16,18]. Differences in study design may also play a role. Enderson et al [23] reported that prospective studies show a higher incidence of missed injuries as compared with retrospective reviews. Patients with clinically significant missed injuries comprise around 15% to 22.3% of total number of patients with missed injuries. Different studies have used different definitions to determine clinical significance. Some publications focused on those missed injuries that were associated with high morbidity and mortality as a result of a delayed diagnosis [1,5]. Others used the requirement of further surgical procedures as criteria to define clinically significant missed injuries [9]. Janjua et al [2] included significant pain, complications, residual disability and death in the definition of a clinically significant missed injury. In general, studies tended to report higher incidence of clinically significant missed injuries if they related the clinical significance to alterations in therapy [5]. In summary, these findings call for more standardized investigations to provide more exact information about the incidence of missed injuries after trauma.

In twenty seven percent of polytrauma patients a pelvic fracture can be detected [24]. Especially in severely injured patients, pelvic instability is associated with severe bleeding [25-31] and undetected pelvic injuries may lead to exsanguination or shock [29]. We observed a decreased incidence in missed pelvic injuries after trauma that has not yet been described. Previous studies have reported limitations of pelvic x-rays in the detection of intra-articular and acetabular fractures [32,33]. However, the widespread availability of Multiple Slice Computed Tomography (MSCT) scans and integration of computed tomography (CT) in the emergency room [34-36] has improved the speed [37,38] and accuracy [37,39-41] of diagnostic procedures and has led to early detection of injuries. Furthermore, since the diagnostics of a critically injured patient must focus on life-threatening injuries, the pelvis is usually scanned as part of combined abdomen/pelvis CT examination [37,42]. That also allows for an early detection of pelvic injuries. Less significant extremity injuries are usually detected upon further examinations [7].

When the publications carried out a classification of missed injuries (minor injuries, major injuries, life threatening injuries), we observed that approximately 27–66% of unrecognized injuries were major injuries. These injuries are potentially clinically significant factors for morbidity and mortality. Several studies demonstrated that trauma patients with missed injuries and delayed diagnoses required significantly longer hospital stays (15.7–42.1 days vs. 7.9–26.7 days) and longer intensive care unit stays (5.4–10.9 days vs. 1.5–5.7 days), than those without missed injuries [5-8]. Some studies report high rates of mortality [1,6,8,9,22] among trauma patients with missed injuries. A possible relationship between delay of diagnoses and morbidity was reported in one study [3].

### Strategies to limit missed injuries

Thorough clinical and radiological examinations represent the main tools for the diagnosis of fractures and injuries. While clinical examination of awake and alert patients leads to the diagnosis of clinically significant missed injuries, further diagnostic methods (radiologic imaging) continue to be beneficial in unconscious patients [42-44]. Several studies report lack of admission radiographs of the specific area of injury (46.3–53.8%) [14,18] and misinterpreted x-rays (15–34.9%) [1,5] as main radiological factors contributed to missed diagnosis. Further factors are clinical inexperience (26.5%) [19] and assessment errors (33.8–60.5%) [1,2,5,6]. Other investigations found additional contributing factors such as technical errors [2], inadequate x-rays [5,19,21], interrupted diagnosis [17], and neighbouring injuries [1]. Authors [2,18], however, noted that patients with missed injuries and delayed diagnoses tend to have a combination of contributing factors. Janjua et al [2] found that in 50% of cases, more than one factor was responsible.

To reduce the rate of missed injuries, we must focus on unconscious and intubated patients with severe trauma (ISS↑) and brain injuries (GCS↓) during the primary and secundary survey [1-3,5-9]. Furthermore, some authors emphasized the role of tertiary trauma survey in patients with multiple injuries, as significant injuries may be missed during the primary and secondary surveys [2,3,6,9]. Approximately fifty percent of overall missed injuries and ninety percent of clinically significant missed injuries were diagnosed by tertiary trauma survey within 24 hours of admission [2,3]. However, this survey can also be performed after the patient has gained consciousness and is able to voice complaints, or before discharge from the intensive care unit [6]. The tertiary trauma survey (TTS) should cover: (1) standardized re-evaluation of blood tests, (2) careful review initial x-rays, and (3) clinical assessment for the effective detection of occult injuries. Furthermore, as musculoskeletal injuries are usually missed during the first and second survey, an experienced orthopaedic surgeon must be involved in the tertiary survey.

## Conclusion

Missed injuries still occur at an unacceptably high rate in trauma patients. Standardization of tertiary survey will lead to a decrease in missed injuries and an improvement in patient outcome. Therefore, this survey is vitally important and should be a part of trauma care. Furthermore, the lack of standardized studies that use comparable definitions of missed injuries and clinically significant missed injuries calls for further investigations to produce more reliable data.

## Competing interests

The authors declare that they have no competing interests.

## Authors' contributions

All authors were involved in the research project and preparation of the manuscript. PHC: He made a substantial contribution to conception and design, and gave a critical and final approval. PR: He has collected the data and made an analysis and interpretation of these data. He also made a draft of the manuscript and revisions. All authors read and approved the final version of the manuscript.
